# Editorial: Challenges and current research status of vertigo/vestibular diseases, volume II

**DOI:** 10.3389/fneur.2024.1409139

**Published:** 2024-05-02

**Authors:** Jun Wang, Andrea Castellucci, Hubertus Axer, Sulin Zhang

**Affiliations:** ^1^Department of Otorhinolaryngology, Union Hospital, Tongji Medical College, Huazhong University of Science and Technology, Wuhan, China; ^2^ENT Unit, Department of Surgery, Azienda USL-IRCCS, Reggio Emilia, Italy; ^3^Department of Neurology, Jena University Hospital, Jena, Germany

**Keywords:** vertigo, vestibular disorders, benign paroxysmal positional vertigo (BPPV), bilateral vestibulopathy (BV), Meniere's disease, persistent postural-perceptual dizziness (PPPD)

Vertigo and vestibular diseases are prevalent among middle-aged and older adults, posing an increased risk of falls and thereby significantly contributing to injury and disability. These conditions also exert a notable impact on individuals' psychological wellbeing. Management can be particularly challenging, as symptoms are often nonspecific and may indicate various underlying causes. The ten published articles in this Research Topic encompass clinical and basic research on common vertigo disorders, as well as reviews and case reports.

This topic includes two articles on Benign Paroxysmal Positional Vertigo (BPPV), which is the most common cause of vertigo. Chen X. et al. conducted a comparative analysis between the modified Epley maneuver and the traditional method for treating posterior semicircular canal BPPV. Their research unveiled that, compared to the traditional method, the modified Epley maneuver significantly enhanced repositioning success rates and reduced instances of canal switching. Previous research has suggested a link between BPPV and various mental disorders, but due to methodological constraints, the results remain contentious. Therefore, Liu et al. employed the Mendelian randomization method to investigate the association between BPPV and seven mental disorders. They did not find a significant causal relationship between BPPV and bipolar disorder, depression, anxiety disorder, schizophrenia, or suicidal tendencies. But they conclude that neuroticism and mood swings may be risk factors for BPPV.

There are two articles on Bilateral Vestibulopathy (BVP) in this Research Topic. BVP is known for its diverse clinical manifestations and multiple underlying causes, contributing to its heterogeneous and chronic nature. van Stiphout et al. conducted a narrative review focusing on key insights and advancements concerning the clinical presentation of BVP. Additionally, they introduced a novel diagnostic algorithm and discussed current and prospective therapeutic approaches. Moreover, variability exists in the reported presence or absence of Vestibular Evoked Myogenic Potentials (VEMPs) among studies involving BVP patients. Lucieer et al. showcased that multi-frequency testing yielded a higher incidence of otolith responses. Cervical VEMPs (cVEMPs) were more frequently present than ocular VEMPs (oVEMPs). Notably, the majority of present VEMP responses were detected during testing at 500, 750, and 1,000 Hz, with fewer occurrences at 2,000 Hz, which also exhibited significantly higher thresholds (for cVEMPs).

Additionally, three articles focus on the application of nystagmus in this topic. Spontaneous nystagmus (SN) serves as a prevalent clinical indicator of peripheral vestibular disorders (1, 2). It commonly manifests as horizontal or horizontal-torsional, direction-fixed movements, accentuated by the removal of visual fixation. Furthermore, its slow phase velocity (SPV) adheres to Alexander's law, wherein the nystagmus amplitude intensifies when the eye moves in the direction of the fast phase (2). Huang et al. elucidated the three-dimensional features of nystagmus elicited by various semicircular canal combinations in healthy young individuals. Additionally, they established an initial reference range for the SPV and its asymmetry in nystagmus induced by the vertical semicircular canal. This research serves to deepen understanding of the mechanisms underlying semicircular canal-induced nystagmus and enhance the diagnostic precision of nystagmus in patients with otogenic vertigo. Zhang et al. demonstrated a positive correlation between the SPV of horizontal and torsional components of SN in vestibular neuritis (VN) patients and the angular vestibulo-ocular reflex (aVOR) gain asymmetry observed in the video head impulse test (vHIT). Additionally, they found that the direction of SN aligns with the plane of the affected semicircular canals. Superior VN typically presents with horizontal-vertical upward-torsional nystagmus, while total VN exhibits horizontal nystagmus with a prominent torsional component but no vertical component. Weak nystagmus, observed upon fixation removal, may occur in both normal individuals and during recovery from unilateral vestibular disturbances, yet its clinical relevance remains unclear in patients experiencing dizziness. Chen C-C. et al. conducted a comparison of nystagmus characteristics at different stages following unilateral vestibular loss. Their findings suggest that nystagmus with visual fixation may diminish as early as 1 week after the onset of acute unilateral vestibular loss. Conversely, nystagmus in the absence of visual input persisted at a subdued level for several months, with its direction predominantly aligning with the anticipated pattern of paralytic nystagmus.

In a case report, MRI follow-ups depicted the evolving transition from an initial inflammatory response to the development of endolymphatic hydrops within the inner ear (Chen Y. et al.). Chen Y. et al. detailed these progressive changes, observed from labyrinthitis to endolymphatic hydrops, as visualized in inner ear MRI scans of a patient diagnosed with Meniere's disease (MD) and suspected immune dysfunction. This visual representation highlights the correlation between MD and inflammation, offering valuable insights into its pathogenesis to inform treatment decisions. Qi et al. demonstrated that employing postauricular injection of nitroglycerin provides a safer and more effective approach for modeling migraine in rats compared to intraperitoneal injection. This novel method of establishing a migraine animal model not only confirms the impact of migraine on hearing but also establishes a groundwork for future clinical research.

Persistent postural-perceptual dizziness (PPPD) typically arises following conditions that disrupt balance or manifest as acute or episodic vertigo, unsteadiness, or dizziness (Yagi et al.). An essential characteristic of PPPD warranting further investigation is its exacerbation upon exposure to moving or complex visual stimuli. Yagi et al. discovered that vestibular inputs may not be fully integrated within the vestibulo-visuo-somatosensory network. They observed an increase in functional connectivity (FC) between visuospatial and spatial cognitive areas even in healthy individuals following visual stimuli. Therefore, these results showing heightened FC from visual areas to spatial cognitive and prefrontal areas subsequent to visual stimuli may contribute to explaining the prolonged symptoms experienced after visual exacerbation in PPPD.

In a nutshell, this Research Topic, consisting of 10 articles covering diverse subjects within the realm of vertigo or vestibular diseases, unveiled the significant potential of this area of research and facilitated advancements in this challenging field of study.

## Author contributions

JW: Writing – original draft. AC: Writing – review & editing. HA: Writing – review & editing. SZ: Writing – review & editing.
